# COVID-19 ICU mortality prediction: a machine learning approach using SuperLearner algorithm

**DOI:** 10.1186/s44158-021-00002-x

**Published:** 2021-09-01

**Authors:** Giulia Lorenzoni, Nicolò Sella, Annalisa Boscolo, Danila Azzolina, Patrizia Bartolotta, Laura Pasin, Tommaso Pettenuzzo, Alessandro De Cassai, Fabio Baratto, Fabio Toffoletto, Silvia De Rosa, Giorgio Fullin, Mario Peta, Paolo Rosi, Enrico Polati, Alberto Zanella, Giacomo Grasselli, Antonio Pesenti, Paolo Navalesi, Dario Gregori, for the VENETO ICU Network, Martina Tocco, Chiara Pretto, Enrico Tamburini, Davide Fregolent, Pier Francesco Pirelli, Davide Marchesin, Matteo Perona, Nicola Franchetti, Michele Della Paolera, Caterina Simoni, Tatiana Falcioni, Alessandra Tresin, Chiara Schiavolin, Aldo Schiavi, Sonila Vathi, Daria Sartori, Alice Sorgato, Elisa Pistollato, Federico Linassi, Sara Gianoli, Silvia Gaspari, Francesco Gruppo, Alessandra Maggiolo, Elena Giurisato, Elisa Furlani, Alvise Calore, Eugenio Serra, Demetrio Pittarello, Ivo Tiberio, Ottavia Bond, Elisa Michieletto, Luisa Muraro, Arianna Peralta, Paolo Persona, Enrico Petranzan, Francesco Zarantonello, Alessandro Graziano, Eleonora Piasentini, Lorenzo Bernardi, Roberto Pianon, Davide Mazzon, Daniele Poole, Flavio Badii, Enrico Bosco, Moreno Agostini, Paride Trevisiol, Antonio Farnia, Lorella Altafini, Mauro Antonio Calò, Marco Meggiolaro, Francesco Lazzari, Ivan Martinello, Francesco Papaccio, Guido di Gregorio, Alfeo Bonato, Camilla Sgarabotto, Francesco Montacciani, Parnigotto Alessandra, Giuseppe Gagliardi, Gioconda Ferraro, Luigi Ongaro, Marco Baiocchi, Vinicio Danzi, Paolo Zanatta, Katia Donadello, Leonardo Gottin, Ezio Sinigaglia, Alessandra da Ros, Simonetta Marchiotto, Silvia Bassanini, Massimo Zamperini, Ivan Daroui, Walter Mosaner

**Affiliations:** 1 Unit of Biostatistics, Epidemiology and Public Health, Department of Cardiac, Thoracic, Vascular Sciences, and Public Health, University of Padova, Padova, Italy; 2 Department of Medicine (DIMED), Padova University Hospital, Padova, Italy; 3 Institute of Anaesthesia and Intensive Care Unit, Padova University Hospital, Padova, Italy; 4 Anaesthesia and Intensive Care Unit, Ospedale Riuniti Padova Sud, Schiavonia, Italy; 5 Anaesthesia and Intensive Care Unit, Ospedale di San Donà di Piave e Jesolo, San Donà di Piave, Italy; 6 Anaesthesia and Critical Care Unit, San Bortolo Hospital, Vicenza, Italy; 7 Anaesthesia and Intensive Care Unit, Ospedale Dell’Angelo, AULSS 3 Serenissima, Mestre, Italy; 8 Anaesthesia and Intensive Care Unit, Ospedale Ca’ Foncello, AULSS 2 Marca Trevigiana, Treviso, Italy; 9 Emergency Medical Services, Regional Department, AULSS 3, Venice, Italy; 10 Anaesthesia and Intensive Care Unit B, Department of Surgery, Dentistry, Gynaecology and Pediatrics, University of Verona, AOUI - University Hospital Integrated Trust, Verona, Italy; 11 Anaesthesia and Critical Care, Department of Pathophysiology and Transplantation, University of Milan, Milan, Italy; 12 Department of Anaesthesia, Intensive Care and Emergency Medicine, Fondazione IRCCS Ca’ Granda-Ospedale Maggiore Policlinico, Milan, Italy

**Keywords:** COVID-19, Machine learning, ICU, Mortality, Predictive model

## Abstract

**Background:**

Since the beginning of coronavirus disease 2019 (COVID-19), the development of predictive models has sparked relevant interest due to the initial lack of knowledge about diagnosis, treatment, and prognosis. The present study aimed at developing a model, through a machine learning approach, to predict intensive care unit (ICU) mortality in COVID-19 patients based on predefined clinical parameters.

**Results:**

Observational multicenter cohort study. All COVID-19 adult patients admitted to 25 ICUs belonging to the VENETO ICU network (February 28th 2020-april 4th 2021) were enrolled. Patients admitted to the ICUs before 4th March 2021 were used for model training (“training set”), while patients admitted after the 5th of March 2021 were used for external validation (“test set 1”). A further group of patients (“test set 2”), admitted to the ICU of IRCCS Ca’ Granda Ospedale Maggiore Policlinico of Milan, was used for external validation. A SuperLearner machine learning algorithm was applied for model development, and both internal and external validation was performed. Clinical variables available for the model were (i) age, gender, sequential organ failure assessment score, Charlson Comorbidity Index score (not adjusted for age), Palliative Performance Score; (ii) need of invasive mechanical ventilation, non-invasive mechanical ventilation, O_2_ therapy, vasoactive agents, extracorporeal membrane oxygenation, continuous venous-venous hemofiltration, tracheostomy, re-intubation, prone position during ICU stay; and (iii) re-admission in ICU.

One thousand two hundred ninety-three (80%) patients were included in the “training set”, while 124 (8%) and 199 (12%) patients were included in the “test set 1” and “test set 2,” respectively. Three different predictive models were developed. Each model included different sets of clinical variables. The three models showed similar predictive performances, with a training balanced accuracy that ranged between 0.72 and 0.90, while the cross-validation performance ranged from 0.75 to 0.85. Age was the leading predictor for all the considered models.

**Conclusions:**

Our study provides a useful and reliable tool, through a machine learning approach, for predicting ICU mortality in COVID-19 patients. In all the estimated models, age was the variable showing the most important impact on mortality.

**Supplementary Information:**

The online version contains supplementary material available at 10.1186/s44158-021-00002-x.

## Background

Predictive modeling has been a hot topic of coronavirus disease 2019 (COVID-19) research [1]. Since the very beginning of the epidemic, there was a significant push towards developing predictive models for COVID-19 diagnosis and prognosis. The interest in predictive models' development was associated with the initial lack of knowledge about COVID-19 diagnosis/treatment/prognosis and the unexpected and dramatic pressure on the healthcare system, especially on intensive care units (ICU) [2]. Such predictive models were aimed at helping physicians stratify patients’ risk of developing the outcome of interest, e.g., need of hospitalization and mechanical ventilation.

A systematic review of the literature by Wynants et al. identified more than sixty predictive models already published at the beginning of the pandemic, i.e., April 2020. The update of this systematic review recorded more than two hundred models [1]. Initially, most models focused on COVID-19 diagnosis, while the update of the revision showed that much more published models focused on patients' prognosis and particularly on predicting death risk.

The idea behind such algorithms was to characterize patients at higher risk of death from severe acute respiratory syndrome coronavirus 2 (SARS-CoV-2) infection to help physicians identify the best treatment for each patient according to his/her characteristics. The final aim was to guarantee an efficient allocation of the healthcare resources given the dramatic shortage resulting from the outbreak.

Italy was the first European country hit by the COVID-19 outbreak. Lombardy and Veneto were the two Italian regions where COVID-19 spread first. In a short time, healthcare authorities tried to activate emergency measures to contain the virus spread at the population level and organize the healthcare system response to face the sudden and unexpected increased demand for healthcare assistance [2–5]. In the Veneto Region, the “COVID-19 VENETO ICU Network” was established [5]. It is an official task force aimed at optimizing ICU resources management through the identification of dedicated COVID-19 pathways and the increase of ICU beds capacity. Furthermore, the network aims to share experience on COVID-19 patients’ treatment among intensive care medicine specialists to standardize patient care. Finally, data on COVID-19 patients admitted to the COVID-19 ICUs of the network have been collected routinely, allowing the epidemiological surveillance of the phenomenon, e.g., to plan the activation of additional ICU beds and clinical research.

The aim of the present study was to develop and validate a predictive model through a machine learning approach for ICU mortality in COVID-19 patients using VENETO ICU Network data.

## Methods

We prospectively screened the records of all adult patients with confirmed SARS-CoV-2 infection admitted to the ICUs of the COVID-19 VENETO ICU network, between 28th of February 2020 and 4th of April 2021 [5, 6]. COVID-19 diagnosis was made according to the World Health Organization interim guidance (http://www.who.int/docs/default-source/coronaviruse/clinical-management-of-novel-cov.pdf).

The study was approved by the Institutional Ethical Committee of each participating center (coordinator center approval reference number 4853AO20) and informed consent was obtained for each patient in compliance with national regulation and the recommendations of the Institutional Ethical Committee of Padova University Hospital.

The study cohort was divided into two groups, according to the time of ICU admission. The first group, i.e., “training set,” included patients admitted to the ICUs from 28th of February to 28th of April 2020 plus from 27th of November to 4th of March 2021, and was used for model training. The second group (named “test set 1”), composed by patients admitted to the ICUs from 5th of March to 4th of April 2021, was used for external validation of the model.

In addition to that, a third group (named “test set 2”), composed by patients admitted to the ICU of IRCCS Ca’ Granda Ospedale Maggiore Policlinico of Milan (Lombardy Region) in the same period of time, was also used for external validation.

At ICU admission, the physicians in charge of the patients prospectively collected a predefined set of clinical variables at ICU admission, as listed in Supplementary Materials (Table S1), and entered data into a predesigned data collection form implemented in a web-based system. Moreover, the physicians recorded the need of respiratory support, tracheostomy, re-intubation, prone positioning, extracorporeal membrane oxygenation, continuous venous-venous hemofiltration, vasoactive agents during ICU stay, or re-admission. Each investigator had a personal username and password. Patients’ privacy was protected by assigning a de-identified patient code. Prior to data analysis, two independent investigators and a statistician screened the database for errors against standardized ranges and contacted local investigators with any queries. Then, validated data were entered in the database for final analysis.

### Models estimation

Three SuperLearner (SL) prediction tools were developed and validated on the ICU data (see Additional file 1, Table S1, for the complete list of variables included in each model)

1. Model 1. The first model was tuned considering only the variables collected at ICU admission having less than 85% of missing data (see Additional file 1, Table S1, for the complete list of variables included in the model). The external validation was performed on the “test set 1 and 2.”
2. Model 2. The second model was tuned considering all the variables collected at ICU admission, even though missing data were more than 85% (Additional file 1, Table S1). The external validation was performed on the “test set 1.”
3. Model 3. The third model was tuned considering the variables collected at ICU admission and during ICU stay, even though the missing data were more than 85% (Additional file 1, Table S1). The external validation was performed on the “test set 1.”

### SuperLearner approach

**Fig. 1:**
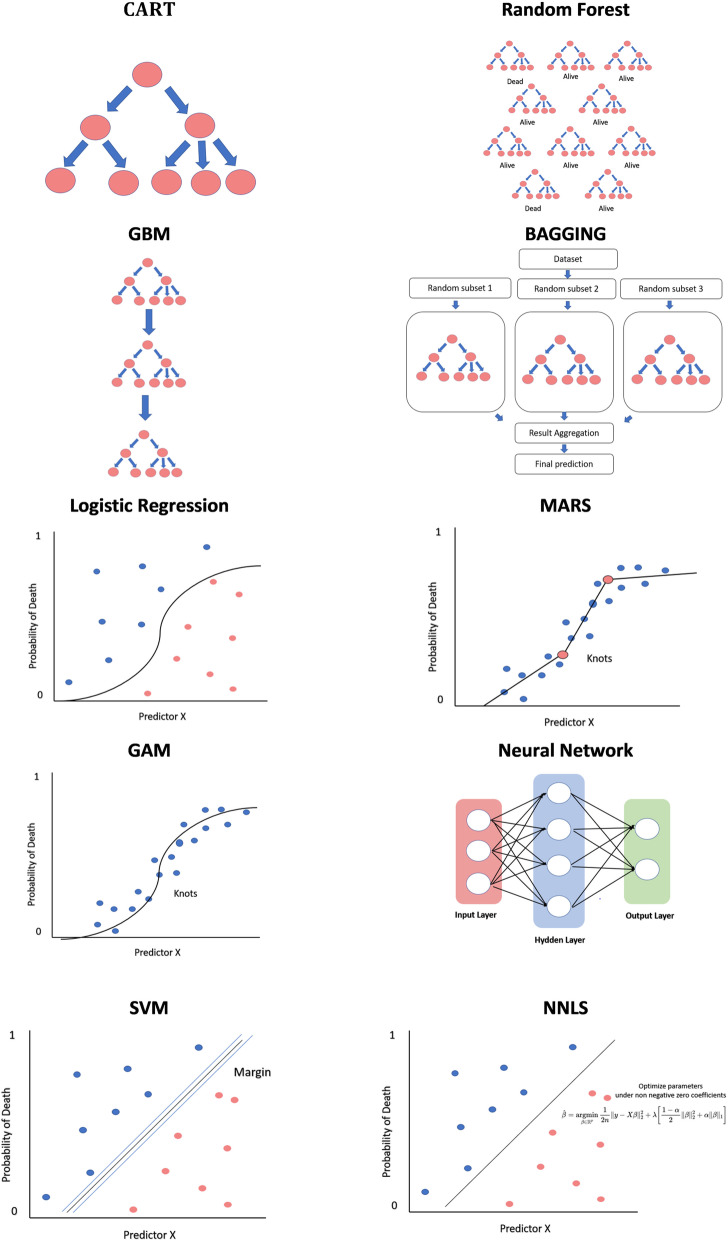
Schematic representation of the base learners used in the Super Learner ensemble model

SuperLearner (SL) is an ensemble Machine Learning algorithm that combines multiple Machine Learning Techniques (MLTs), i.e., base learners, to achieve the best possible weighted performance of the base learners [7–9]. The detailed description of the algorithms is provided in the Additional file 1, Methods S2. Figure 1 presents the schematic representations of the base learners.

### Performance measures

The sensitivity, specificity, F1 statistics, the balanced accuracy, and were computed. The training ROC plots were reported.

### Internal cross-validation

The base models and the SuperLearner models underwent internal cross-validation performing a 5-fold cross-validation procedure.

### Variable importance plot

The variable importance plots were reported. The importance measure was computed considering the mean decrease in the ROC measure resulting from the removal of the variable within the permutations, as recommended in the literature [10].

### Descriptive statistics

Continuous data were reported I quartile/median/III quartile categorical data were reported as a percentage and absolute frequencies.

### Shiny web application

A shiny web application was developed. The tool calculates the ICU death probability, according to the patients’ characteristics based on each one of the models estimated.

## Results

### Study population

The overall population included 1616 patients. The first 1293 (80%) patients admitted to the ICUs of the VENETO ICU Network were used for models training (“training set”), while the following 124 (8%) patients were used for external validation (“test set 1”). As well, a further cohort of 199 (12%) patients, admitted to the IRCCS Ca’ Granda Ospedale Maggiore Policlinico, was used as additional external validation (“test set 2”).

Table 1 presents the training and validation cohorts’ characteristics. The proportion of deaths was of 39% in the cohort of 1417 patients admitted to the ICUs of the VENETO ICU Network, and 28% in the cohort of 199 patients admitted to the IRCCS Ca’ Granda Ospedale Maggiore Policlinico.

**Table 1:** Training and test sets characteristics

	“Training set” Model 1 (*n* = 1293)	“Training set” Models 2–3 (*n* = 656)	“Test set 1” (COVID-19 VENETO ICU Network) (*n* = 124)	“Test set 2” (IRCCS Ca’ Granda Ospedale Maggiore Policlinico) (*N* = 199)
Gender (m)	78% (1002)	78% (506)	68% (84)	75% (150)
Age	60/69/75	61/68/74	56/67/73	51/61/66
**SOFA score**	3/4/7	3/4/6	4/6/8	4/5/7
**Individual components of SOFA score**				
SOFA score: assessment of respiratory system (PaO2/FiO2): 0	5% (50)	1% (7)	0% (0)	1% (2)
1	1% (16)	2% (13)	2% (2)	1% (2)
2	29% (324)	44% (278)	44% (48)	8% (16)
3	34% (372)	28% (179)	29% (32)	48% (94)
4	31% (337)	24% (153)	24% (26)	42% (83)
SOFA Platelets (×10^3^/μL): 0	90% (567)	90% (567)	52% (58)	
1	6% (38)	6% (38)	21% (23)	
2	3% (18)	3% (18)	27% (30)	
3	0% (3)	0% (3)	0% (0)	
4	1% (4)	1% (4)	0% (0)	
SOFA Glasgow Coma Scale (GCS): 0	63% (400)	63% (400)	69% (75)	
1	23% (148)	23% (148)	25% (27)	
2	3% (17)	3% (17)	3% (3)	
3	1% (7)	1% (7)	2% (2)	
4	9% (60)	9% (60)	1% (1)	
SOFA bilirubin (mg/dl or umol/L): 0	90% (571)	90% (571)	99% (108)	
1	7% (46)	7% (46)	1% (1)	
2	1% (9)	1% (9)	0% (0)	
3	1% (4)	1% (4)	0% (0)	
4	0% (2)	0% (2)	0% (0)	
SOFA creatinine (mg/ml or umol/L): 0	82% (511)	82% (511)	86% (93)	
1	13% (81)	13% (81)	10% (11)	
2	3% (18)	3% (18)	4% (4)	
4	2% (10)	2% (10)	0% (0)	
SOFA mean arterial pressure or administration of vasoactive agents required: 0	68% (429)	68% (429)	83% (91)	
1	7% (41)	7% (41)	1% (1)	
2	4% (23)	4% (23)	0% (0)	
3	15% (93)	15% (93)	11% (12)	
4	7% ( 41)	7% (41)	5% (5)	
**Palliative Score (PS)**	70/100/100	70/100/100	60/90/100	
**Individual components of PS**				
PS Ambulation: 30	2% (14)	2% (14)	0% (0)	
40	0% (3)	0% (3)	0% (0)	
50	2% (12)	2% (12)	2% (2)	
70	14% (84)	14% (84)	21% (25)	
100	82% (501)	82% (501)	78% (93)	
PS Activity: 50	2% (13)	2% (13)	0% (0)	
60	2% (12)	2% (12)	2% (2)	
80	11% (64)	11% ( 64)	8% (10)	
90	30% (177)	30% (177)	39% (46)	
100	55% (323)	55% (323)	51% (60)	
PS Self-Care: 30	1% (9)	1% (9)	0% (0)	
40	2% (12)	2% (12)	0% (0)	
50	3% (17)	3% (17)	2% (3)	
60	14% (84)	14% (84)	27% (32)	
100	80% (491)	80% (491)	71% (85)	
PS Intake: 20	2% (13)	2% (13)	0% (0)	
30	3% (16)	3% (16)	10% (12)	
80	5% (29)	5% (29)	5% (6)	
100	90% (550)	90% (550)	85% (101)	
PS level of consciousness (LOC): 10	1% (7)	1% (7)	0% (0)	
40	1% (7)	1% (7)	0% (0)	
60	4% (26)	4% (26)	10% (12)	
100	93% (573)	93% (573)	90% (108)	
**Charlson Comorbidity Index (CCI): 0**	48% (616)	44% (291)	66% (82)	60% (119)
**CCI distribution when > 0**	1/2/3	1/2/3	1/2/3	1/1/2
**Individual components of CCI**				
Liver disease: None	96% (1246)	96% (631)	98% (121)	
Mild	2% (20)	2% (14)	2% (3)	
Moderate-severe	2% (27)	2% (11)	0% (0)	
Diabetes mellitus: None or diet-controlled	78% (1012)	80% (522)	82% (102)	
Uncomplicated	10% (127)	15% (96)	8% (10)	
End-organ damage	12% (154)	6% (38)	10% (12)	
Tumor: None	93% (1203)	94% (617)	96% (119)	
Localized	6% (77)	5% (35)	2% (3)	
Metastatic	1% (13)	1% (4)	2% (2)	
Acute myocardial infarction: Yes	12% (153)	11% (73)	3% (4)	
Peripheral vascular disease: Yes	13% (167)	15% (98)	13% (16)	
Chronic heart failure: Yes	8% (99)	8% (50)	4% (5)	
Transient ischemic attack: Yes	4% (54)	4% (23)	1% (1)	
Dementia: Yes	2% (32)	1% (8)	1% (1)	
Chronic obstructive pulmonary disease: Yes	10% (132)	11% (71)	2% (3)	
Connective tissue disease: Yes	3% (34)	4% (24)	2% (2)	
Peptic ulcer disease: Yes	2% (25)	1% (6)	0% (0)	
Hemiplegia: Yes	1% (15)	1% (9)	1% (1)	
Leukemia: Yes	1% (14)	1% (6)	0% (0)	
Chronic kidney disease: Yes	8% (104)	9% (56)	4% (5)	
Lymphoma: Yes	1% (14)	1% (8)	0% (0)	
AIDS: Yes	0% (4)	0% (2)	0% (0)	
**ICU stay**				
O_2_ therapy	8% (104)	16% (104)	29% (36)	
Non-invasive ventilation	48% (626)	44% (286)	43% (53)	
Invasive mechanical ventilation	85% (1093)	79% (521)	61% (76)	
Prone position	62% (797)	57% (374)	64% (79)	
Tracheostomy	17% (226)	15% (101)	2% (3)	
Re-intubation	52% (38)	0% (0)	100% (2)	
Extracorporeal membrane oxygenation	3% (39)	3% (17)	1% (1)	
Continuous venous-venous hemofiltration	6% (73)	5% (30)	2% (3)	
Vasoactive agents	48% (621)	41% (268)	25% (31)	
Re-admission in ICU	1% (9)	1% (9)	0% (0)	

Continuous variables are I quartile/median/III quartile and categorial variables are percentages (absolute numbers)

Model 1 was trained on the overall ‘training set’ of patients (Table 1) because the model included a limited set of variables measured at ICU admission (Supplementary materials, Table S1) with less than 85% of missing data. Models 2 and 3 were trained on 656 out of 1293 patients because they also included variables with more than 85% of missing data (Table 1) (see Supplementary materials, Table S1, for the complete list of variables included in each model). The main difference between Model 2 and Model 3 is that Model 2 included only variables measured at ICU admission, while Model 3 included variables recorded at admission and also during the ICU stay.

Model 1 was validated on both the cohort of 124 patients belonging to the “test set 1” and on the cohort of 199 patients named “test set 2.” Models 2 and 3 were validated on the external cohort of 124 patients admitted to the ICUs of the COVID-19 VENETO ICU Network (“test set 1”).

### Models’ performance

The three models showed similar performances in predicting ICU mortality (Table 2 and Additional file 1, Figure S3), with a training balanced accuracy that ranged between 0.72 and 0.90.

**Table 2:** Training and test validation performances

		Sens	Spec	Balanced accuracy	***F***	ROC
**Model 1**	Training performance	0.74	0.74	0.72	0.77	0.73
	External validation performance (Veneto cohort)	0.69	0.68	0.68	0.76	0.65
	External validation performance (Lombardia cohort)	0.70	0.69	0.70	0.78	0.70
**Model 2**	Training performance	0.78	0.77	0.78	0.79	0.85
	External validation performance	0.67	0.65	0.66	0.76	0.65
**Model 3**	Training performance	0.90	0.89	0.90	0.91	0.93
	External validation performance	0.84	0.83	0.83	0.88	0.81

*Sens* Sensitivity, *Spec* Specificity, Balanced Accuracy, F1 statistics, and ROC are reported for each model

**Fig. 2:**
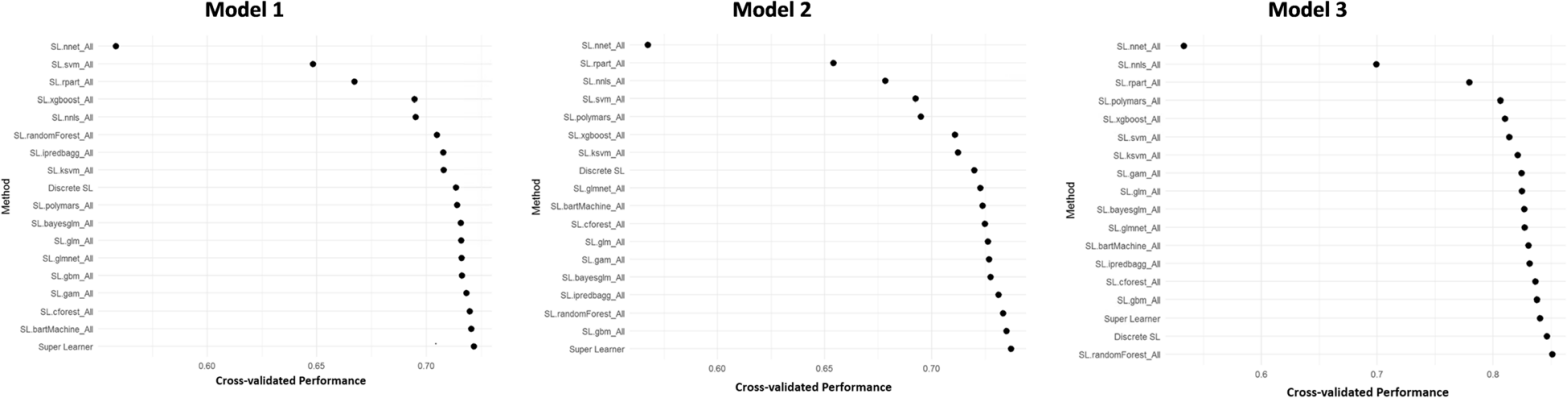
Cross-validated performances. The figure presents cross-validated area under the ROC curves according to base learners and SuperLearner for the three models

The cross-validation performance is in Fig. 2. The best performance was achieved by Model 3, with a ROC of 0.85, while both Models 1 and 2 presented a ROC value of 0.75.

With regards to the performance of the algorithms on which the SuperLearner was based, the RF was the one with the best performance on Model 3, as well as for Model 2, together with the GBM. For Model 1, the best performance was achieved by a Bayesian Machine Regression Trees (BartMachine) (Fig. 2).

### Variables importance in relation to the outcome

**Fig. 3:**
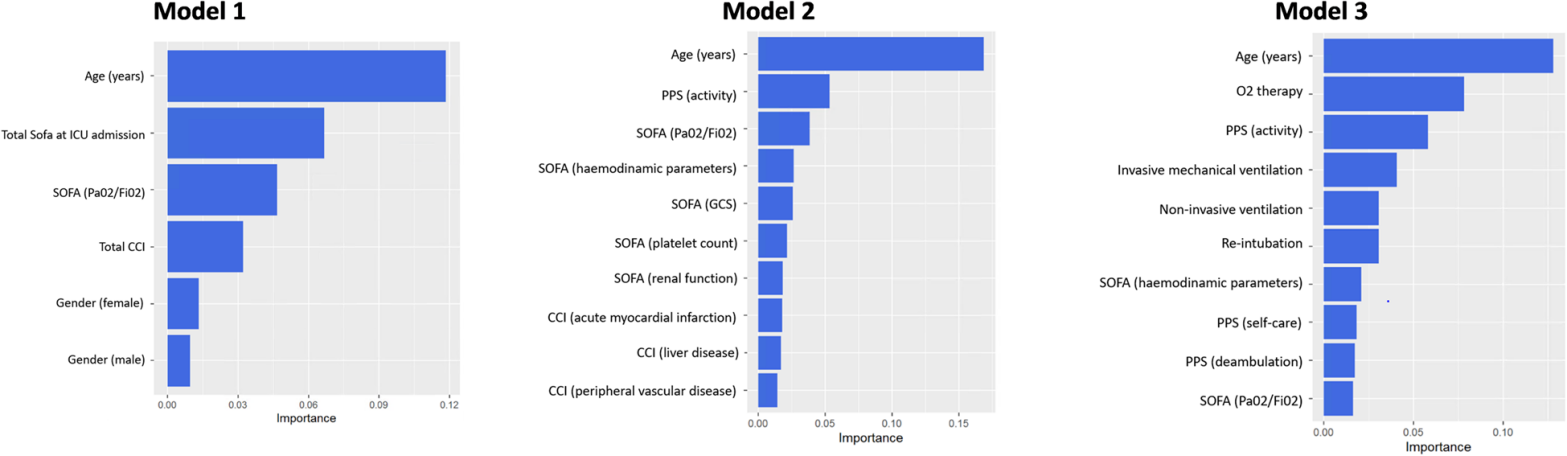
Variable importance plots. The ten most important predictors are reported in the plots. Abbreviations: PaO_2_/FiO_2_, arterial partial pressure of oxygen to inspired oxygen fraction ratio; SOFA, Sequential Organ Failure Assessment; CCI, Charlson Comorbidity Index; GCS, Glasgow Coma Scale; PPS, Palliative Performance Score.

Age was the leading predictor for all the considered models, followed by total SOFA score at ICU admission and the arterial partial pressure of oxygen to inspired oxygen fraction ratio used for SOFA calculation (SOFA PaO_2_/FiO_2_) in Model 1. The SOFA PaO_2_/FiO_2_ was a relevant predictor for Model 2, as well the Palliative Predictive Score (PPS) Activity variable. The PPS Activity was also in the top five parameters for Model 3, together with the need of O2 therapy, non-invasive or invasive ventilation (Fig. 3).

The shiny app reporting the three ICU mortality prediction tools is available at https://r-ubesp.dctv.unipd.it/shiny/CoViD-19%20icupred/.

## Discussion

The present study provides a tool for predicting ICU mortality in COVID-19 patients using data from a large cohort of patients admitted to the ICUs of the COVID-19 VENETO ICU Network. The three models, systematically built through a machine learning approach, showed good training and validation performances, yielding similar results to predict ICU mortality.

In particular, age was identified as the most important predictive parameter in every model investigated. Secondary, total SOFA score at ICU admission, the level of daily activity and the need of different types of respiratory supports were important parameters for Model 1, 2 and 3.

This finding is in line with current literature, describing a great impact of age on mortality in COVID-19 patients undergoing invasive and non-invasive ventilation [6, 11–15]. Karagiannidis C et al, in the widest cohort of hospitalized COVID-19 patients, showed that mortality has been high for patients receiving mechanical ventilation, particularly for patients aged 80 years or older and those requiring dialysis, and has been considerably lower for patients younger than 60 years [11].

Similar findings were reported by Boscolo et al. and Vaschetto et al. investigating in-hospital mortality of COVID-19 mechanically ventilated. In both studies, the cumulative incidence of mortality at 60 days was higher in the older ones [6, 12].

Worth noting, from the beginning of the pandemic, several tools have been proposed for mortality prediction of COVID-19 patients; however, it is difficult to compare their performance because each model was developed on patients with different characteristics, using different sets of variables, and using different techniques for model development. Indeed, Wynants and colleagues have shown that all published models have several limitations [1], including small sample size and lack of information and clarity on algorithm development reporting. For these reasons, it is difficult to compare models’ performance and to identify the most feasible model to be used in everyday clinical practice to assist physicians’ decisions. Our findings show that the SL is a feasible approach to be used with clinical data, providing good predictive performances and good generalizability. Although machine learning approaches are increasingly used in the clinical setting, also in COVID-19 research [7-10], more traditional techniques, i.e., traditional logistic regression for binary outcomes and survival regression models for time-to-event outcomes, are still widely used since they are much simpler to be implemented and interpreted. However, the use of machine learning approaches represents an added value to predictive modeling, as it allows the detection of complex relationships between the outcomes of interest and the covariates, overcoming the limits of traditional analysis, especially when a high number of predictors is evaluated in front of a low number of events.

The predictive tools described in the present paper have several strengths, including the fact that they have been developed on a large multicenter cohort of patients admitted to the ICUs of one of the Italian regions most severely affected by the COVID-19 pandemic, the use of both internal and external validation, and the use of a machine learning tool instead of more traditional techniques to build the predictive model.

However, our study has some limitations. First, clinical variables investigated in our study represent only a small number of parameters potentially relevant and able to affect critically ill patients’ outcomes. Second, several patients had incomplete records, which depended on the overwhelming workload for ICU physicians during the COVID-19 pandemic.

## Conclusions

Our study provides a useful and reliable tool, through a machine learning approach using the SL algorithm, for predicting ICU mortality in COVID-19 patients. Age was the most predictive parameter in all the estimated Models.

## Supplementary Information

**Additional file 1: Table S1.** Variables collected and included in each predictive model. **Methods S2.** SuperLearner algorithm details. **Figure S3.** ROC Curves estimated on the test sample.

## Data Availability

The data that support the findings of this study are available from the corresponding author, PN, upon request.

## Abbreviations

SARS-CoV-2: Severe acute respiratory syndrome coronavirus 2
COVID: Coronavirus disease
ICU: Intensive care unit
PaO_2_/FiO_2_: The ratio between arterial partial pressure of oxygen and inspired fraction of oxygen
SL: SuperLearner
SOFA: Sequential Organ Failure Assessment
MLT: Machine learning technique
ROC: Receiving operative characteristics
CCI: Charlson Comorbidity Index
PPS: Palliative Predictive Score
IMV: Invasive mechanical
OR: Odds ratio
CI: Confidence interval
Sens: Sensitivity
Spec: Specificity
