# Supplementary material for: COVID-19 ICU mortality prediction: a machine learning approach using SuperLearner algorithm
Source: J Anesth Analg Crit Care. 2021 Sep 1;1:3. doi: 10.1186/s44158-021-00002-x (PMC8413709; doi:10.1186/s44158-021-00002-x)
Supplement: Supplementary file 1 — Additional file 1: Table S1. Variables collected and included in each predictive model. Methods S2. SuperLearner algorithm details. Figure S3. ROC Curves estimated on the test sample. [file 44158_2021_2_MOESM1_ESM.docx]

**Additional File 1**

**COVID-19 ICU mortality prediction: a machine learning approach using SuperLearner algorithm.**

Giulia Lorenzoni^1*^, Nicolò Sella^2*^ MD, Annalisa Boscolo^3^ MD, Danila Azzolina^1^, Patrizia Bartolotta^1^, Laura Pasin^3^ MD, Tommaso Pettenuzzo^3^ MD, Alessandro De Cassai^3^ MD, Fabio Baratto MD^4§^, Fabio Toffoletto^5§^ MD,  Silvia De Rosa^6^ MD, Giorgio Fullin MD^7§^, Mario Peta^8§^ MD, Paolo Rosi^9^ MD, Enrico Polati^10§^ MD, Alberto Zanella^11,12^ MD, Giacomo Grasselli^11,12^ MD, Antonio Pesenti^11,12^, Paolo Navalesi^2,3§^ MD, FERS, Dario Gregori PhD^1^ for  the COVID-19 VENETO ICU Network°.

**Table S1. Variables collected and included in each predictive model.**

**Methods S2. Super Learner algorithm details**

**Figure S3. ROC Curves estimated on the test sample.**

**References**

**Table S1. Variables collected and included in each predictive model.**

| **Model** | **Variables** |
| --- | --- |
| **General Database** | Gender, Age, Individual items of the SOFA score, total SOFA score at ICU admission, Individual items of the CCI, total CCI, Individual items of the Palliative Performance Score (1), total Palliative Score, Invasive mechanical ventilation, Noninvasive mechanical ventilation, Conventional O_2_ therapy, Vasoactive agents, Extracorporeal Membrane Oxygenation, Continuous venous-venous hemofiltration, Tracheostomy, Reintubation, Prone position, Re-admission in ICU |
| Model 1 | Gender, Age, total SOFA score, SOFA PaO_2_/FiO_2_, total CCI, |
| Model 2 | Gender, Age, Individual items of the SOFA score, Individual items of the CCI, Individual items of the Palliative Performance Score |
| Model 3 | Gender, Age, Individual items of the SOFA score, Individual items of the CCI, Individual items of the Palliative Performance Score, Invasive mechanical ventilation, Noninvasive mechanical ventilation, Conventional O_2_ therapy, Vasoactive agents, Extracorporeal Membrane Oxygenation, Continuous venous-venous hemofiltration, Tracheostomy, Re-intubation, Prone position, Re-admission in ICU |

SOFA: Sequential Organ Failure Assessment (2); ICU: Intensive Care Unit; PaO_2_/FiO_2_: arterial partial pressure of oxygen to inspired oxygen fraction ratio; CCI: Charlson Comorbidity Index (not adjusted for age) (3).

**S2. Super Learner algorithm details**

To develop a Super Learner algorithm, it is necessary to define a library set of L learners $(\pi_{1}, \ldots,\pi_{L})$, specifying a meta-learning method $\Phi$ and get a partition of the training observation into V-folds (for us V = 5) to carry out the cross-validation for the performance evaluation.

#### Base learner Algorithms

The base learner algorithms considered for the analysis are reported below.

*Classification and Regression Trees (CART)* (4) are methods for constructing prediction models obtained by recursively partitioning the data space and fitting a simple prediction model within each partition. As a result, the partitioning can be represented graphically as a decision tree. The *Conditional tree (CTREE)* has been also considered within the methods. Unlike the classical CART model, CTREE uses a significance test procedure to select variables instead of selecting the variable that maximizes an information measure (e.g. Gini coefficient) (2). The significance test is permutations where the distribution of the test statistic under the null hypothesis is obtained by calculating all possible values of the test statistic under rearrangements of the labels on the observed data points.

*Random Forest (RF)* (5) is a typical ML technique that recursively creates multiple decision trees. It selects a subset of available features and recursively partitions the data in the regression space until the amount of variation in the subspace is small. Random forest is a greedy technique and as a result, it does not necessarily converge to the global optimal solution. To avoid such indecisive convergence, a collection or ensemble of locally optimal trees can be done (bagging.) The ensemble of such trees is known as a forest.

*Bagging Trees* is an ML that falls into the category of ensemble learning. In bagging several CART algorithms are trained on different datasets, each one obtained from the initial dataset through random sampling with replacement (bootstrap). The name bagging derives from the combination of the words bootstrap (that is the random sampling with replacement) and aggregation (referring to the aggregation of more models, typical of ensemble learning) (5).

*Gradient Boosting Machine (GBM)* (6) tree-based model involving a recursive addition to the initial learning from the residuals was applied. It fits a tree-based model on the residuals using the specified list of variables at hand and explains the variance in the residuals. The total number of trees specified for the model building was 500 with interaction depth as 5 and the learning weight of iteration was 0.1.

*Generalized Linear Model (GLM)* with elastic net regularization (7) is a regularized regression method that linearly combines the L1 and L2 penalties of the lasso and ridge methods applied in synergy with a link function a variance function to overcome linear model limitation (such as the constant variability among the mean and the normality of the data). A simple Generalized Linear (GLM) Logistic Regression model has been also considered. The Bayesian version of a GLM (BGLM) model has been also considered for the computation.

*Polychotomous regression or classification based on Multivariate Adaptive Regression Splines (POLYMARS)*(8) uses linear splines and selected tensor products to fit multiple classifications in a way that avoids estimating pure multiple classification methods, i.e. try to estimate $P(Y=k|X$), focusing on the estimation of a reliable conditional class probabilities for the classification.

XGBoost is a decision-tree-based ensemble machine learning algorithm that can be used for classification or regression problems increasing the model accuracy. XGB uses a gradient boosting framework; the idea is to build trees sequentially in such a way as to ensure that the errors of the previous three are reduced; on the contrary, RF combines results at the end of the process training independently each classifier (9).

*Neural Network (NNET)* in the simplest form the Neural Network models are composed of an input layer, a hidden layer, and an output layer (three layers of “neurons” which are connected). The features extracted by the model represent the input layer and are used to predict an output. The nodes displayed in the input layer communicate with each node in the hidden layer which is connected to an output layer. The purpose is to compute a weighted sum based on neurons’ importance and to provide an output (10).

*A generalized additive model (GAM)* has been also considered. It is a generalized linear model in which the response variable depends linearly on unknown smooth functions of some predictor variables, and interest focuses on inference about these smooth functions (11).

The Support Vector Machine (SVM) algorithm’s main objective is to find an optimal hyperplane of the feature’s N-dimensional space (N—the number of variables) that distinctly classifies the data points into a binary partition. Several hyperplanes may separate the classes of data points. The SVM algorithm considers the hyperplanes, which maximize the margin (the distance between data points of classes). The functional form of Hyperplane separators may be linear or smoothed via the Kernel density function. Both parameterizations have been considered (12-14).

A non-negative least squares (NNLS) classifier has been also considered. The idea is that unknown samples can be approximated by sparse non-negative linear combinations of few training samples. Based on sparse coefficient vectors representing the training data, a sparse interpreter can then be used to predict the class label.

*Missing Data Imputation*

SL cannot handle missing data, for this reason, we performed a Multiple Hot Deck Imputation. The technique uses the degree of affinity between the row with missing data and each potential donor row to generate weights such that rows more closely resembling the row with missingness are more likely to be drawn as donors.

**Figure S3. ROC Curves estimated on the test sample.**

| **Model 1**  **AUROC=0.73**  **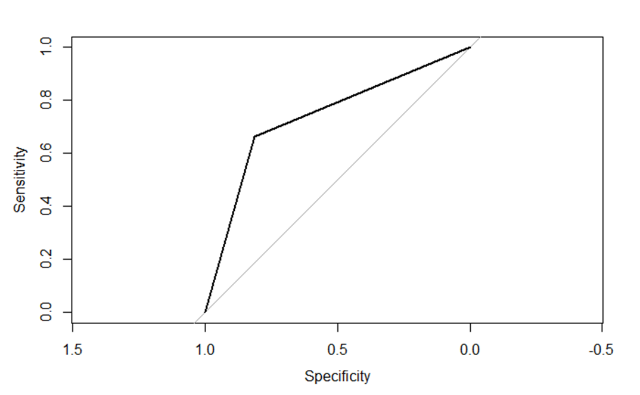** |
| --- |
| **Model 2**  **AUROC=0.85**  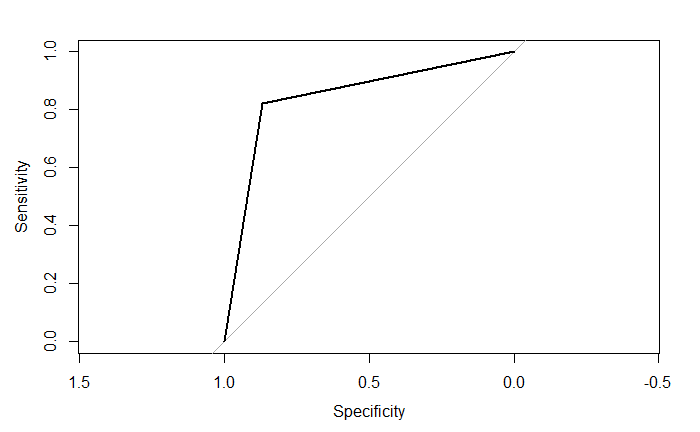 |
| **Model 3**  **AUROC=0.93**  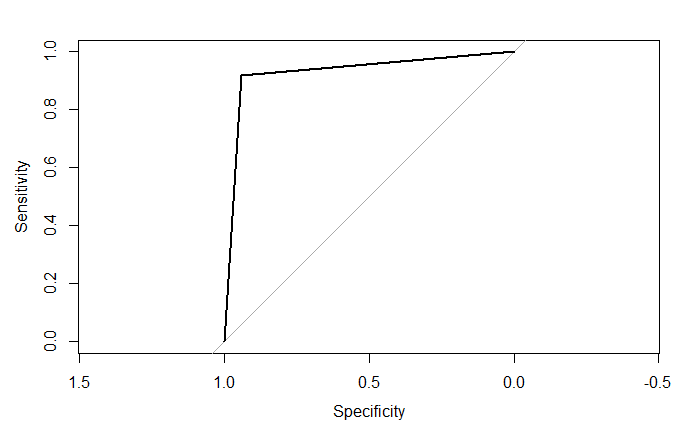 |

The Area under ROC has also been reported (AUROC).

**References**

1. [Anderson F, Downing GM, Hill J, Casorso L, Lerch N. Palliative Performance scale (PPS): a new tool (1996). J Pall Care 12(1):5-11](https://www.ncbi.nlm.nih.gov/pubmed/8857241)

2. [Vincent](https://pubmed.ncbi.nlm.nih.gov/?sort=date&term=Vincent+JL&cauthor_id=8844239) JL, [R Moreno](https://pubmed.ncbi.nlm.nih.gov/?sort=date&term=Moreno+R&cauthor_id=8844239), [J Takala](https://pubmed.ncbi.nlm.nih.gov/?sort=date&term=Takala+J&cauthor_id=8844239), [S Willatts](https://pubmed.ncbi.nlm.nih.gov/?sort=date&term=Willatts+S&cauthor_id=8844239), et al. The SOFA (Sepsis-related Organ Failure Assessment) score to describe organ dysfunction/failure. On behalf of the Working Group on Sepsis-Related Problems of the European Society of Intensive Care Medicine (1996). Intensive Care Med 22(7):707-10

3. [Charlson](https://pubmed.ncbi.nlm.nih.gov/?sort=date&term=Charlson+ME&cauthor_id=3558716) ME, [Pompei](https://pubmed.ncbi.nlm.nih.gov/?sort=date&term=Pompei+P&cauthor_id=3558716) P, [Ales](https://pubmed.ncbi.nlm.nih.gov/?sort=date&term=Ales+KL&cauthor_id=3558716) KL, [MacKenzie](https://pubmed.ncbi.nlm.nih.gov/?sort=date&term=MacKenzie+CR&cauthor_id=3558716) CR (1987). A new method of classifying prognostic comorbidity in longitudinal studies: development and validation. J Chronic Dis 40(5):373-83

4. Breiman L, Friedman JH, Olshen RA, Stone CJ. Classification and Regression Trees (1984). Wadsworth and Brooks/Cole

5. Hothorn T, Bretz F, Westfall P. Simultaneous inference in general parametric models (2008). Biometrical journal 50(3):346–63

6. Breiman L. Random forests (2001). Machine Learning 45(1):5–32

7. Breiman L. Bagging Predictors (1996). Machine Learning 24(2):123–40

8. Friedman JH. Greedy Function Approximation: A Gradient Boosting Machine (2001). The Annals of Statistics 29(5):1189–232

9. Friedman J, Hastie T, Tibshirani R. Regularization Paths for Generalized Linear Models via Coordinate Descent (2010). J Stat Softw 33(1):1–22

10. Kooperberg C, Bose S, Stone CJ. Polychotomous Regression (1997). Journal of the American Statistical Association 92(437):117–27

11. Chen T, Guestrin C. XGBoost: A Scalable Tree Boosting System. In: Proceedings of the 22nd ACM SIGKDD International Conference on Knowledge Discovery and Data Mining [Internet]. San Francisco California USA: ACM; 2016. p. 785–94. Available from: https://dl.acm.org/doi/10.1145/2939672.2939785

12. Bishop CM. Neural networks for pattern recognition (1995). Oxford : New York: Clarendon Press ; Oxford University Press; 1995. 482 p.

13. Hastie T, Tibshirani R. Generalized additive models. Boca Raton, Fla: Chapman & Hall/CRC; 1999. 335 p.

14. Cortes C, Vapnik V. Support-vector networks. Machine Learning. 1995;20(3):273–97.
